# STM-induced light emission from thin films of perylene derivatives on the HOPG and Au substrates

**DOI:** 10.1186/1556-276X-6-347

**Published:** 2011-04-19

**Authors:** Aya Fujiki, Yusuke Miyake, Yasushi Oshikane, Megumi Akai-Kasaya, Akira Saito, Yuji Kuwahara

**Affiliations:** 1 Department of Precision Science & Technology, Graduate school of Engineering, Osaka University, 2-1 Yamada-oka, Suita 565-0871, Japan; 2 PRESTO, Japan Science and Technology Agency (JST), 4-1-8 Honcho, Kawaguchi, Saitama 332-0012, Japan

## Abstract

We have investigated the emission properties of *N,N*'-diheptyl-3,4,9,10-perylenetetracarboxylic diimide thin films by the tunneling-electron-induced light emission technique. A fluorescence peak with vibronic progressions with large Stokes shifts was observed on both highly ordered pyrolytic graphite (HOPG) and Au substrates, indicating that the emission was derived from the isolated-molecule-like film condition with sufficient π-π interaction of the perylene rings of perylenetetracarboxylic diimide molecules. The upconversion emission mechanism of the tunneling-electron-induced emission was discussed in terms of inelastic tunneling including multiexcitation processes. The wavelength-selective enhanced emission due to a localized tip-induced surface plasmon on the Au substrate was also obtained.

## Introduction

Control of molecular emission from organic materials has attracted much attention owing to its potential applications not only in basic molecular science but also in research on soft material devices such as organic light-emitting diodes (OLEDs) and biosensors [1-4]. Scanning-tunneling-microscope-induced light emission (STM-LE) spectroscopy is highly effective for characterizing the optical and electronic properties of nanoscale materials such as organic single molecules or thin films at the atomic scale. However, it involves serious analytical difficulties in receiving extremely weak signals from the objective materials. To overcome such difficulties, it is promising to combine STM-LE spectroscopy with plasmon enhancement on surfaces. Surface plasmons at the interface between metallic and dielectric media generate an intense electromagnetic field on the surface, which provides an efficient enhancement field for some optical processes such as the fluorescence/phosphorescence emission and optical absorption of organic materials on a metal surface [1]. We have first observed the fluorescence of Cu phthalocyanine under enhancement utilizing an STM-tip-induced plasmon (TIP) [5]. For light emission from single molecules, Qiu et al. [6] reported light emission from individual Zn(II)-etioporphyrin I molecules adsorbed on Al_2_O_3_/NiAl(110), in which an oxide buffer layer is used to prevent fluorescence quenching and disturbance of pronounced plasmon emission [7-9]. They explained that the spectra were due to the de-excitation of excited anion states resulting from hot electron injection. The plasmon enhancement effect is also expected to be applied to the development of light-emitting diodes [2,10]. Recently, we have developed a high-efficiency OLED including Au nanoparticles owing to the enhancement effect of localized surface plasmons on metal nanostructures [10].

Perylenetetracarboxylic diimide (PTCDI) and its derivatives are n-type semiconductors [11,12], used in various optoelectronic devices such as thin-film transistors [13], photovoltaic [14], and light-emitting diodes [15]. PTCDI molecules have been expected as a material of single-molecule devices [16] because of the high thermal and photostabilities of PTCDI. In this study, we have studied the STM-LE from *N,N*'-diheptyl-3,4,9,10-perylenetetracarboxylic diimide (PTCDI-C7) thin films on HOPG and Au substrates. We elucidated the intrinsic optical properties of PTCDI-C7 in terms of the STM-LE spectra on the HOPG substrate compared with the absorption and photoluminescence (PL) spectra, and demonstrated the wavelength control of enhanced molecular luminescence, i.e., the selective enhancement of the resonant wavelength of PTCDI-C7 through TIP enhancement effects on the Au substrate. We also discussed the emission mechanism of upconversion fluorescence.

## Experimental

PTCDI-C7 was synthesized by a modification of a previously reported method [17,18]. A freshly cleaved HOPG and Au thin films evaporated on mica were used as the substrates. PTCDI-C7 thin films were prepared by spin-coating 0.4 mg/ml PTCDI-C7 solution in 1-tetradecene at a spin velocity of 1000 rpm, followed by rinsing with the solvent and drying in vacuum desiccators for 24 h. The film thickness was about 5-10 nm, which was determined by comparing the PL intensities of the PTCDI-C7 thin films fabricated by the spin coating method with those fabricated by evaporation in vacuum with thicknesses of 5, 10, 15, and 20 nm, which were estimated using a thickness monitor. STM (Digital Instruments Co. Ltd., USA, Nanoscope IIIa) measurement was carried out at room temperature under ambient conditions and a mechanically sharpened Pt/Ir tip was used. The collected photons were guided to a photomultiplier tube (Hamamatsu Photonics, Japan, R-649S) using an optical fiber to obtain a light intensity map (the dark count was less than 1 count per second (cps) at 253 K; the wavelength detection range was 300-850 nm). To acquire optical spectra, a grating spectrometer (Roper Scientific, USA, SpectraPro-300i) with a liquid-N_2_-cooled charge-coupled device camera (Roper Scientific, USA, Spec-10:100B/LN; the detection range was 200-1100 nm) was employed. The absorption and photoluminescence (PL) spectra of PTCDI-C7 were obtained using a UV-visible/NIR spectrophotometer (Hitachi High-Technologies Co., Japan, U-3010) and a custom-built system with an argon-ion laser (Edmond Optics, USA, Multi-Line 150 mW) at 514 nm, respectively.

## Results and discussion

**Figure 1:**
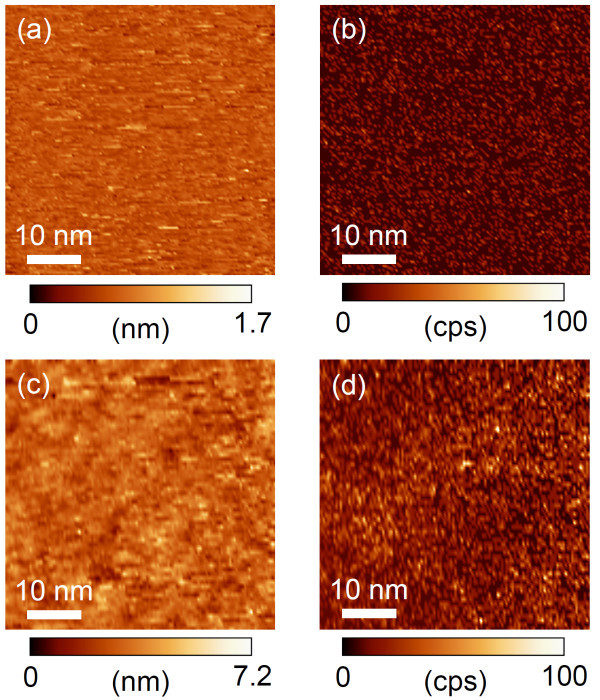
**STM topographic images and photon integration maps of PTCDI-C7 thin films**. STM topographic images on (a) HOPG and (c) Au substrates, and photon integration maps on (b) HOPG and (d) Au substrates. Pairs of a topographic image and a photon map ((a) and (c), (b) and (d)) were obtained simultaneously (Vs = +2.2 V, It = 20 nA).

Figure 1a,c shows STM topographic images of the PTCDI-C7 thin films on the HOPG and Au substrates, and Figure 1b,d shows photon intensity maps corresponding to the STM images in Figure 1a,c, respectively. These pairs of topographic and photon images were obtained simultaneously in the constant-current mode. The surface roughnesses of the molecular films in Figure 1a,c were induced by the surface morphologies of the pristine substrates: The surface of the HOPG substrate was atomically flat and that of the as-deposited Au substrate showed a relatively large corrugation. In both the substrates, it was found that the molecules are not well crystallized but show an amorphous behavior. Homogeneous emissions were observed from the entire scanned area in both Figure 1b,d, so that homogeneous and smooth PTCDI-C7 thin films were formed on both the substrates, which showed a good correspondence of the STM topographic images. In the STM-LE measurement in this study, the tip was placed in contact with the thin film under our high-current condition; as a result, the tips might have swept molecules during the scan, in which tunneling electrons directly passed through the thin film to the substrate without an air gap between the tip and the sample.

**Figure 2:**
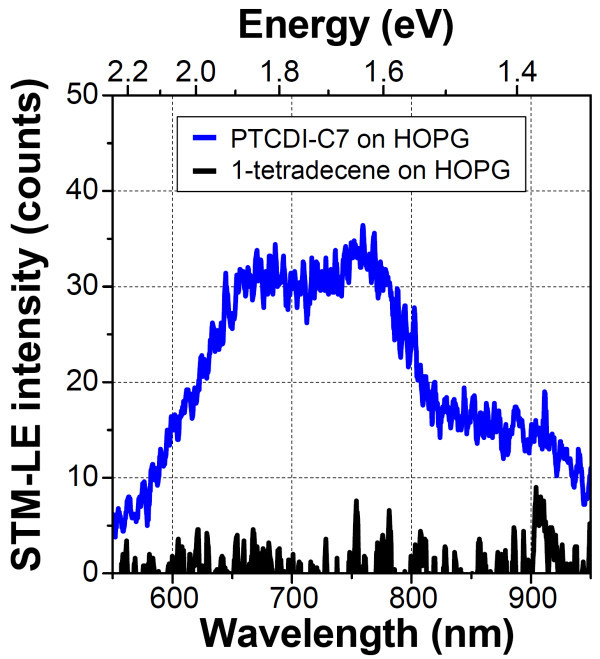
**STM-LE spectra of PTCDI-C7 thin film (blue line) and solvent molecules (black line) on HOPG substrate (Vs = +2.2 V, It = 20 nA, acquisition time = 15 min)**. Both spectra are smoothened by averaging the 10 nearest points of the raw data.

Figure 2 shows the STM-LE spectrum obtained from the PTCDI-C7 thin film (blue line) on the HOPG substrate. The spectrum shown in black represents the result of a 1-tetradecene (solvent) thin film without PTCDI-C7 molecules on the HOPG substrate. In both the spectra, the sample bias voltage, tunneling current, and accumulation time were fixed at +2.2 V, 20 nA, and 15 min, respectively. Both the spectra were acquired with the tip scanning 50 × 50 nm^2 ^of the surface. No emission was observed from the 1-tetradecene thin film; in contrast, sufficient emission was observed from the PTCDI-C7 thin film on the HOPG substrate. To the best of our knowledge, there are only a few STM-LE studies of the HOPG substrate, since there is no surface plasmon mode on the HOPG surface in the visible light wavelength region and plasmon enhancement cannot be effectively used to obtain meaningful STM-LE intensities from adsorbed molecules. We considered that the sufficient intensity of the STM-LE from the PTCDI-C7 thin film on the HOPG substrate is caused by a high quantum yield of the radiative decay of PTCDI-C7 (93% [19]). Uehara and Ushioda [20] reported the STM-LE of a single molecule of rhodamine 6G adsorbed on the HOPG surface. In their study, the quantum yield of light emission via the transition of an electron from the lowest unoccupied molecular orbital to the highest occupied molecular orbital was also high (95% [21]). Note that we obtained no light emission from the PTCDI-C7 thin film fabricated by deposition in vacuum on the HOPG surface, suggesting that the morphology of molecular thin films affected by fabrication processes affects the emission efficiency in STM-LE. A strong visible light is radiated by TIP on the metal substrates such as Au, Ag, and Cu. TIP emission is superimposed on the emission from the adsorbed molecules, so that it is difficult to extract the true spectra of target molecules on metal surfaces. Thus, the STM-LE spectra of adsorbed molecules on an HOPG substrate with no plasmon resonance in the visible spectral range can be used to analyze the intrinsic molecular emission without any disturbance of TIP emission, although the interaction of the molecules with the HOPG surface must be taken into account.

**Figure 3:**
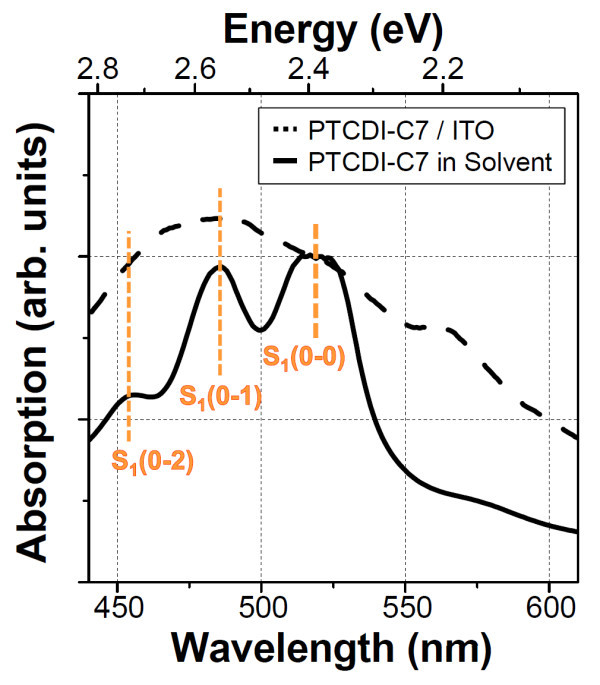
**Absorption spectra of PTCDI-C7 dissolved in 1-tetradecene (0.4 mg/ml) (solid line) and of PTCDI-C7 thin film fabricated using the spin coating method on ITO (dashed line), whose intensities are normalized at 520 nm wavelength**.

Figure 3 shows the absorption spectra of PTCDI-C7 dissolved in 1-tetradecene (0.4 mg/ml, solid line) and of the PTCDI-C7 thin film fabricated on an indium-tin oxide (ITO) substrate using the spin coating method (dashed line), in which the same method of sample preparation as that for the PTCDI-C7 thin film on the HOPG substrate was employed. In the spectrum of PTCDI-C7 solution, we found three distinct peaks at 455, 485, and 520 nm. These peaks are attributed to the S_1_(0-0) transition (S_1 _is a first singlet excited state of PTCDI-C7, numbers in parentheses denote the vibronic levels in the initial and final states) and its vibronic progressions with an energetic distance between the peaks of approximately 0.18 eV. The excitation energy from the ground (S_0_) state to the S_1 _state of PTCDI and its derivatives is 2.36 eV [22], and the energy intervals of the peaks correspond to the energy of the benzene-ring stretch oscillation of perylene (0.15 eV [23]). The obtained absorption spectrum of PTCDI-C7 solution was in good agreement with those in a previous report on perylene derivatives such as *N,N*'-dimethyl-PTCDI and *N,N*'-bis(2,6-xylyl)-PTCDI in dilute solutions by Schouwink et al. [24]. It is considered that the spectrum of PTCDI-C7 solution in Figure 3 is governed by monomer absorption and not ascribed to dimers or larger aggregates [25], which could be a result of the relatively long alkane substituents of PTCDI-C7 that prevent their aggregation through their steric effect. For the PTCDI-C7 thin film, in contrast, the spectrum became highly broadened with an additional small peak at 565 nm compared with that of PTCDI-C7 solution. The peak broadening and the emergence of the new peak are caused by the strong π-π interaction within molecular aggregates, and by the formation of dimers [22,24] or a crystal phase [24,25] due to the strong molecular stacking between PTCDI skeletons, respectively.

**Figure 4:**
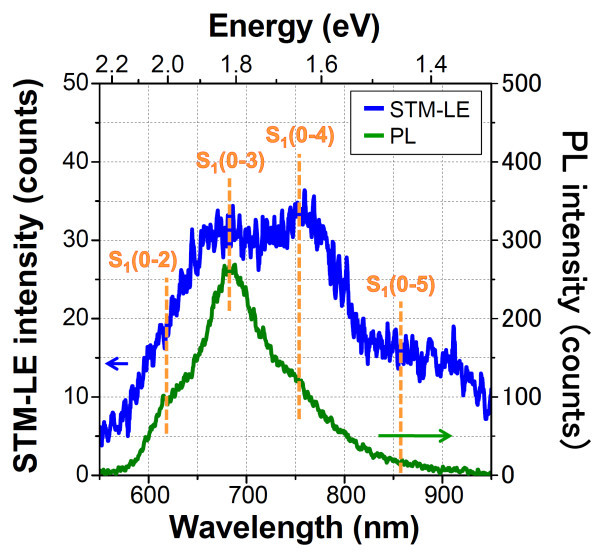
**Photoluminescence spectrum excited by an Ar-Ne laser at 514 nm (green line) and STM-LE spectrum (blue line) of PTCDI-C7 thin film on HOPG substrate (Vs = +2.2 V, It = 20 nA, acquisition time = 15 min)**.

Figure 4 shows the PL spectrum of the PTCDI-C7 thin film on the HOPG substrate (green line). The STM-LE spectrum of the PTCDI-C7 thin film on the HOPG substrate is shown in blue in the figure. It was found that the PL spectrum had a pronounced peak at 680 nm and shoulders at 625 and 750 nm. The obtained peaks of the PL spectrum were ascribed to the vibronic progressions related to the S_1_(0-0) transition at 520 nm, as shown in the absorption spectra in Figure 3, because the energy intervals of the observed PL peaks were approximately 0.17 eV corresponding to the stretching energy of perylene rings, as mentioned earlier. The peaks at 625, 680, and 750 nm were assigned to the S_1_(0-2), S_1_(0-3), and S_1_(0-4) transitions with respect to the S_1 _state, respectively. The PL spectrum included a large Stokes shift of approximately 100 nm compared with the absorption spectra. Note that the PL spectra of the PTCDI-C7 thin films on the ITO and HOPG substrates almost coincided with each other in terms of peak shape and position (data not shown), indicating that the electronic configurations, which are related to the optical properties of the PTCDI-C7 thin films on the ITO and HOPG substrates, were similar to each other. In the STM-LE spectrum of the PTCDI-C7 thin film, some pronounced peaks were observed at 550-950 nm. The peaks of STM-LE were explained by the vibronic progressions related to the S_1 _transition because the peak positions in the PL and STM-LE spectra almost coincided with each other. One can see that the STM-LE spectrum has a broad band up to 900 nm and that the peaks including higher indexes of progressions (up to the S_1_(0-5) transition at 860 nm) are more discriminable than those of the PL spectrum. This result would be derived from our STM-LE condition, e.g., with a local electric field between the STM tip and the substrate surface or with structural deformation of the molecules scratched by a scanning STM tip, which affects the transition probability of electronic excitation or radiation.

Our interest in both PL and STM-LE spectra was aroused by our observation of distinct vibronic progressions, similar to the case of the isolated molecular condition, even in the thin-film configuration of PTCDI-C7 where a moderate intermolecular interaction appeared on the fluorescence spectra in the form of a large (approximately 100 nm) Stokes shift. We assumed that PTCDI-C7 molecules had a poorly crystalline orientation/distribution in the thin film fabricated by the spin coating method due to the steric effect of long alkane substituents, which led them to have a quasi-isolated molecular condition in the thin film structure in terms of the perylene-ring-stretching vibration, although proper π-π stacking exhibiting a large Stokes shift and peak broadening in the spectra of the thin film structures remained. How to extend the fact that the electronic configurations of the PTCDI-C7 molecules are modified by the distribution in the thin film, such as induction, conjugation, and electrostatic, remains controversial. To evaluate such electronic effects, other experiments, such as photoemission spectroscopy and scanning tunneling spectroscopy, should be required.

**Figure 5:**
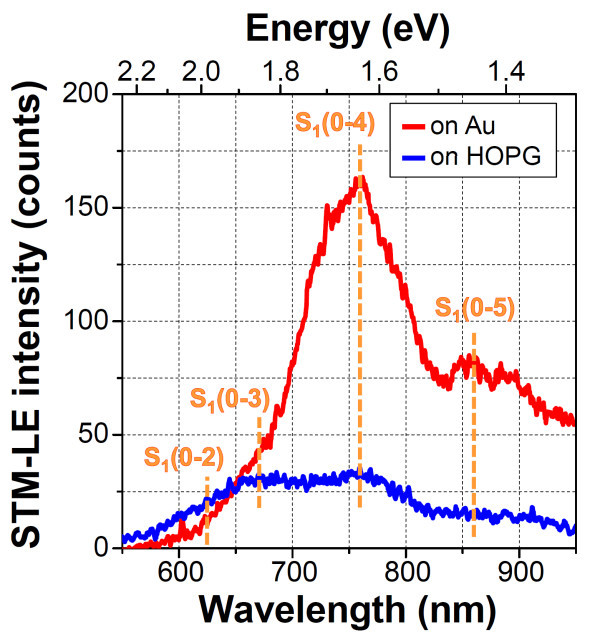
**STM-LE spectra of PTCDI-C7 thin films on Au (red line) and HOPG (blue line) substrates (Vs = +2.2 V, It = 20 nA, acquisition time = 15 min)**.

Figure 5 shows the STM-LE spectra of the PTCDI-C7 thin films on the Au (red line) and HOPG (blue line) substrates. Two spectra were obtained under the same STM conditions (Vs = +2.2 V, It = 20 nA). The emission on the Au substrate originated from the PTCDI-C7 molecules because the peak positions for the Au substrate were consistent with those for the HOPG substrate. It should be noticed that the emission intensities of the peaks at 750 and 860 nm were significantly enhanced about fivefold, whereas the peak intensities at 625 and 680 nm were unchanged. Such a selective enhancement of the emission peaks can be explained by the resonance matching with the TIP mode on the Au substrate. In general, the wavelength of the emission by TIP strongly depends on both the material and shape of the metal tip/substrate. In our case, the resonance wavelength of TIP characterized by the Pt/Ir tip and Au substrate was located in the wavelength range of 700-1000 nm [26]. We clearly showed that TIP selectively enhances emission peaks related to vibronic transitions that are energy-matched to the resonance wavelength of TIP. Thus far, photoluminescence measurements of molecular thin films related to surface plasmon enhancement effects have been carried out. They have shown that molecular fluorescence/phosphorescence intensities are significantly enhanced on noble-metal surfaces [27,28]; however, it is difficult to control the selective enhancement on metal surfaces because the wavelength of surface plasmons varies over a wide band owing to the nanoscale and random roughness of actual metal surfaces. For the selective enhancement of molecular emission, the resonance energy for the fluorescence/phosphorescence of luminescent layers and their associated surface plasmon excitation mode should be adjusted using size- and shape-controlled metal nanoparticles [10,29]. Ino et al. [30] observed STM-LE luminescence from one of the perylene derivatives (i.e., 3,4,9,10-perylenetetracarboxylic dianhydride: PTCDA) deposited on a Ag(111) surface. They found that not only molecular emission but also plasmon-mediated emission is quenched in the case of 1 ML PTCDA adsorption owing to the hybridization of the surface electronic state and the modification of the dielectric constant of the STM gap. In the 2 ML PTCDA thin film, however, they observed one broad structureless peak of molecular fluorescence. The behavior of the STM-LE of PTCDI-C7 obtained in this study differed from their results, which might be due to the morphology of the thin films used.

**Figure 6:**
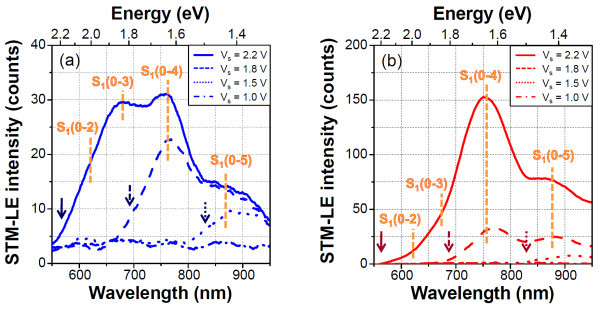
**Bias voltage dependences of STM-LE spectra of PTCDI-C7 thin films on (a) HOPG and (b) Au substrates (It = 20 nA, acquisition time = 15 min)**. The arrow indicates the quantum cutoff energy (see text) of each sample voltage. The spectra are smoothened by averaging the 100 nearest points of the raw data.

To discuss the mechanism of STM-LE emission from PTCDI-C7 in more detail, we determined the sample bias voltage dependence of the spectra of PTCDI-C7. Figure 6a,b shows the variation in the emission spectra as a function of the sample bias voltages on the HOPG and Au substrates, respectively. The arrows indicate the wavelengths of the quantum cutoff energies converted from the corresponding bias voltages. It was considered that the emission from the PTCDI-C7 thin film on the HOPG surface was excited by inelastic tunneling [31] because no polarity dependence of the STM-LE spectra was observed (data not shown), indicating that the injection-type electron-hole recombination mechanism, as in an OLED, is impossible.

The most surprising result in terms of the excitation mechanism in this study was that the sample bias voltage (the energy of tunneling electrons) of all the observed STM-LE emissions shown in Figure 6 did not satisfy the excitation energy of the S_1_(0-0) transition of 2.36 eV. Currently, it is difficult to precisely clarify the excitation mechanism. To realize the obtained phenomena, a total emission process must contain (i) an upconversion process, (ii) a novel excited state (S'_1_) energetically lower than the S_1 _state, and (iii) an initial S_0 _state of molecular excitation consisting of higher vibrational states of PTCDI-C7 (following the electronic excitation of S_0_(n) → S_1_(0)). (i) In the first scheme, multielectron/multistep excitation processes should be introduced; however, these multiexcitation processes must be excluded because of the low quantum efficiency of inelastic tunneling [32], which is also supported by the sample bias dependence of the STM-LE results (Figure 6) in which all of the emissions satisfied the cutoff condition (hν ≤ eVs). The triplet-triplet annihilation (TTA) mechanism enhanced by TIP (we observed the TTA fluorescence in Cu phthalocyanine thin films on the Au substrate [5]) could not be accepted since we observed sufficient intensity of the emission on the HOPG substrate and the free-base PTCDI has a low intersystem crossing probability from the singlet state to the triplet state. (ii) In the second scheme, the molecules are excited to the S'_1 _state derived from an intermolecular interaction due to molecular aggregation in the film. We observed a new peak (565 nm) below the S_1 _state in the absorption spectrum, which was also reported in previous works [22,33]. Note that the energy difference between the S_1 _and S'_1 _states was estimated to be 0.34 eV, which is about twice the energy intervals of vibronic levels, suggesting that a reassignment of the vibronic transitions of the observed peaks is required. (iii) The third scheme of the emission mechanism should include, e.g., thermally assisted excitation to the S_0_(n) states and the direct excitation of vibrational levels by inelastic tunneling. Thermal excitation is easily excluded because the excitation of vibrational levels by heat requires a high temperature of >1800 K in the nanocavity of the STM system (*k*T = approximately 0.17 eV), which is refuted by the result of first-principles calculations [34] and the observed molecular stability. Recently, Dong et al. [32] have observed unexpected upconversion electroluminescence such as S_1_(0) → S_0_(n) for porphyrine molecules adsorbed on a Au(111) surface and proposed that the considerable population rate of electrons moving into higher vibrational states in S_0 _state is induced by plasmon-assisted multistep excitation via virtual electronic all excited states in analogy to surface-enhanced Raman scattering. In their case, TIP, excited by both tunneling electrons and plasmon-exciton coupling and acting as a near-field light source, was pumping molecules into higher vibrational excited states of S_0_. In this study, their proposed mechanism could be applied to the emission of the PTCDI-C7 thin film on the Au substrate. We observed a strong sample bias dependence of the peak intensity of the PTCDI-C7 thin films on the Au substrate, i.e., the emission peaks considerably decreased in intensity upon decreasing sample bias voltage in the TIP resonance energy region. However, the above mechanism was hardly accepted in the case of the HOPG substrate because of the lack of assistance from TIP in the observed energy range. This suggests that the plasmon-assisted *direct *vibrational excitation of the ground state S_0 _occurs in the case of the HOPG substrate, since the surface plasmon energy of the HOPG surface is approximately 60 meV [35] and the energy of TIP generated between the HOPG surface and the Pt/Ir tip covers the excitation energy of vibronic levels of approximately 0.17 eV. In either case, the overall excitation and radiation perspectives remain controversial and theoretical support for the STM-LE mechanism is highly required.

## Conclusion

We have investigated the STM-LE from a PTCDI-C7 thin film on HOPG and Au substrates fabricated by spin coating. On the HOPG substrate, we obtained significantly high-emission intensity from the PTCDI-C7 thin films in spite of the lack of the TIP enhancement effect. In the comparison with those of the absorption and PL spectra, the peaks of the STM-LE spectra were attributed to vibronic progressions of the S_1_(0-0) transition. Using the Au substrate, the emission intensities of the higher index of vibronic peaks, whose energy matched the energy of TIP, were selectively enhanced compared with those in the case of the HOPG substrate. The emission mechanism of the upconversion STM-LE for the PTCDI-C7 thin films could be interpreted by the inelastic tunneling including the multiexcitation of the S_0 _states on both HOPG and Au substrates. Such a selective enhancement of molecular emission is quite useful for various applications of OLEDs, plasmonic devices, ultrasensitive sensors, and other devices, through the control of radiative transitions via an intense plasmon enhancement effect.

## Abbreviations

cps: count per second; HOPG: highly ordered pyrolytic graphite; ITO: indium-tin oxide; OLEDs: organic light-emitting diodes; PTCDI: perylenetetracarboxylic diimide; PL: photoluminescence; STM-LE: scanning-tunneling-microscope-induced light emission; TIP: tip-induced plasmon; TTA: triplet-triplet annihilation.

## Competing interests

The authors declare that they have no competing interests.

## Authors' contributions

AF and YK conceived of the idea, designed the study, and drafted the manuscript. AF carried out the experiments and analyzed the data. YM synthesized PTCDI-C7 and gave suggestions on the preparation of the sample. YO participated in the experimental setup. MA-K and AS participated in the analysis of results. All authors read and approved the final manuscript.
